# Primary Jejunal Angiosarcoma

**DOI:** 10.14309/crj.0000000000001818

**Published:** 2025-08-27

**Authors:** Michelle Mai, Sapana Gupta, Kanhai Farrakhan, Shaolei Lu, Paul Akerman, May Min

**Affiliations:** 1 Warren Alpert Medical School of Brown University, Providence, RI; 2 Division of Gastroenterology, Rhode Island Hospital, Providence, RI; 3 Department of Pathology and Laboratory Medicine, Rhode Island Hospital, Providence, RI

**Keywords:** small bowel angiosarcoma, jejunal angiosarcoma

## Abstract

Primary jejunal angiosarcoma is an aggressive and rare soft-tissue neoplasm with a poor prognosis. Multiple modalities such as endoscopic intervention, radiographic imaging, and immunohistochemistry are often used to confirm the diagnosis. Only 62 cases have been reported in the literature of primary jejunal angiosarcoma, and the majority of these patients had distant metastases at initial staging. This is a case of a 51-year-old man who presented with melena found to have a jejunal mass and diagnosed with angiosarcoma with no metastases detected on initial staging. The diagnosis of angiosarcoma was confirmed with histomorphology and immunohistochemistry. Despite early tumor staging and successful small bowel resection, 6 months postsurgical resection patient later presented with metastatic. This case report highlights the importance of a comprehensive diagnostic strategy and raises consideration for earlier systemic therapy in localized neoadjuvant therapy in early-stage small bowel angiosarcoma.

## INTRODUCTION

Angiosarcomas are aggressive, highly vascular, endothelial cell tumors and have a poor prognosis.^1^ They can derive from anywhere in the body due to well-distributed vascular and lymphatic vessels.^2^ The most common presentation involves cutaneous and soft-tissue tumors of the breast, lung, liver, spleen, adrenal glands, and ovaries. Only 62 reported cases of primary small bowel angiosarcoma were described, with the majority involving distant metastases at presentation and a 1-year survival rate of 20.8%.^3^ Multivariate analysis of small bowel angiosarcomas revealed that age, tumor infiltration depth, and multifocal small bowel segment lesions con tribute to worse prognosis. Here we present a unique case of a 51-year-old man diagnosed with jejunal angiosarcoma without metastases that developed recurrence and metastases 6 months after resection. Given the limited literature available on the diagnosis and treatment of small bowel angiosarcoma, this case describes an endoscopic-focused diagnostic strategy and suggests the need for more aggressive management even in early-stage disease.

## CASE REPORT

**Figure 1.**
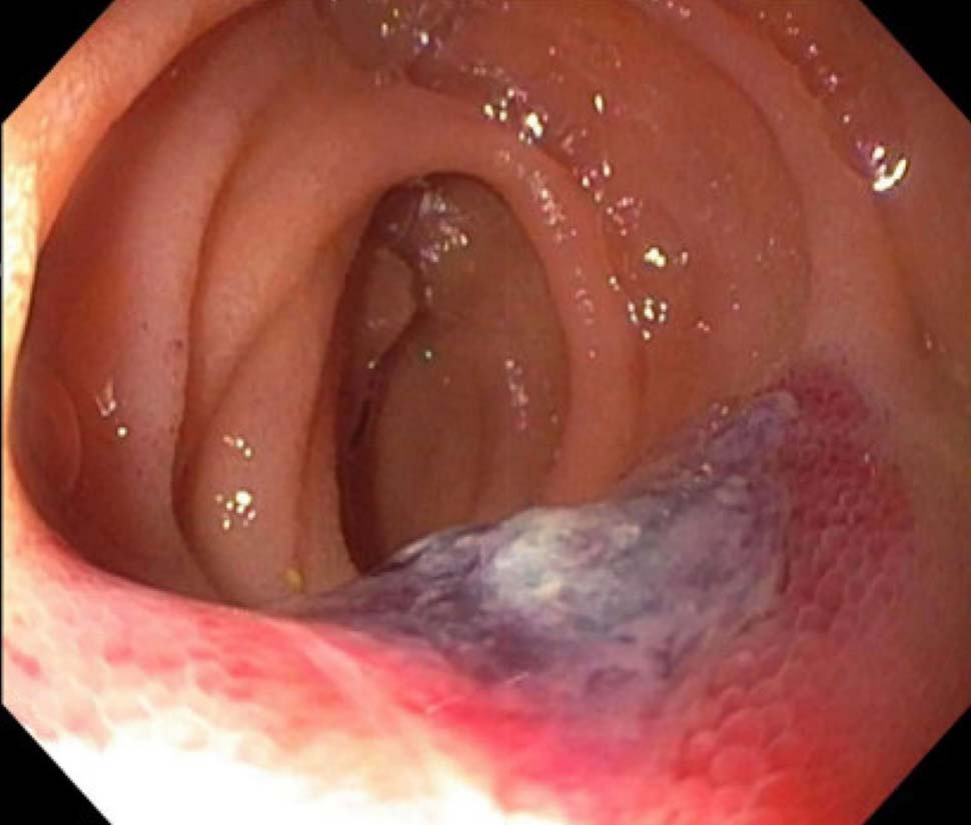
Spiral enteroscopy reveals ulcerated, bleeding jejunal mass of the angiosarcoma.

**Figure 2.**
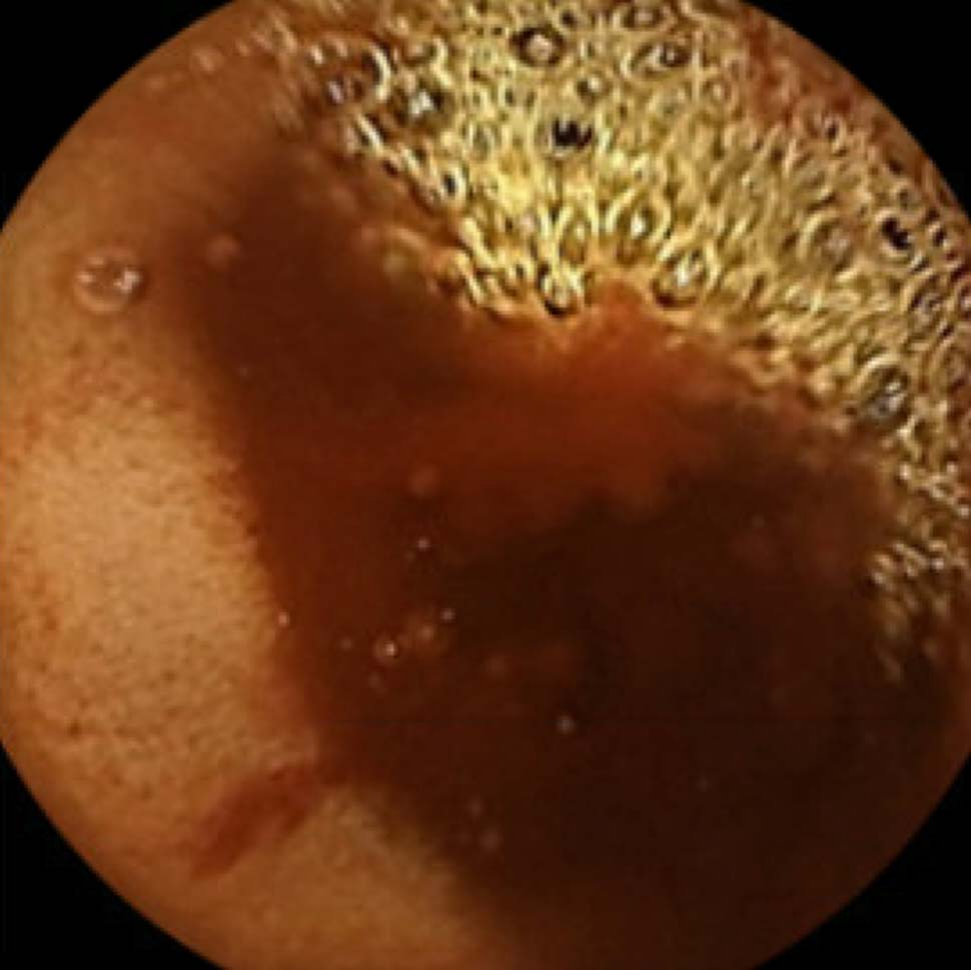
Capsule endoscopy localizes bleeding to small bowel that was presumed to be the jejunum.

A 51-one-year-old man with a medical history of coronary artery disease, non–insulin-dependent diabetes, iron deficiency anemia, and myocardial infarction with coronary stents, on aspirin and clopidogrel, who presented with anemia and dyspnea. Two months earlier, he presented to his primary care physician for dyspnea on exertion and was directed to the nearest emergency department. He had a troponin level of 524 ng/L and hemoglobin 6.2 g/dL. He underwent a cardiac catheterization at an outside hospital which revealed a thrombus and occluded stents. Thrombectomy and percutaneous coronary intervention were unsuccessful, and he was started on ranolazine and discharged. He continued to have worsening dyspnea and new, melanotic stools. Readmission to the same hospital revealed troponin of 719 ng/L, secondary to demand ischemia, and hemoglobin down to 4.3 g/dL from 11.6 g/dL. Esophagogastroduodenoscopy was subsequently performed and revealed Cameron erosions, a 5 cm hiatal hernia, and mild duodenitis without bleeding. A colonoscopy revealed blood in the terminal ileum without a clear source. Small bowel enteroscopy demonstrated a normal-appearing jejunum and duodenum and did not visualize bleeding. The patient's anemia improved with blood transfusion and remained stable after several days of monitoring in the hospital without signs of overt bleeding. Therefore, he was discharged home on a proton pump inhibitor, and his anticoagulation was held with a plan for push enteroscopy as an outpatient (Figures 1 and 2).

**Figure 3.**
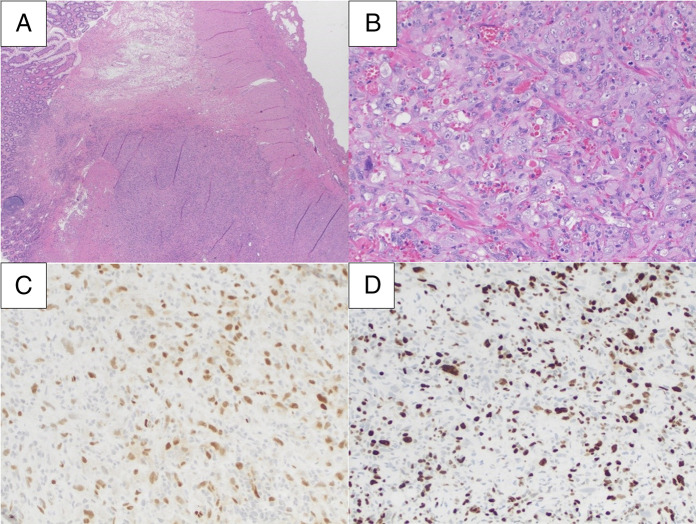
(A) The tumor was mainly located in the lamina propria extending into the muscularis propria (20×). (B) High-power view revealed a vascular tumor with plump, polygonal tumor cells, and pleomorphic nuclei (200×). (C) Endothelial marker, ERG, was diffusely positive (200×). (D) Ki-67 proliferative index was high (∼40%) (200×).

He presented again to the hospital for melanotic stool and outside bloodwork demonstrating hemoglobin of 5.2 g/dL. Upon admission, he was hemodynamically stable and laboratory workup was significant for: hemoglobin 5.4 g/dL, hematocrit 15.7%, iron <10 μg/dL, ferritin 10 ng/mL, and troponin 72 ng/L (peaked at 73 ng/L). On abdominal examination, he was nontender to light and deep palpation in all quadrants. Capsule endoscopy showed active bleeding from the mid small bowel (presumed to be the jejunum). Push enteroscopy did not localize a bleeding source; therefore, a spiral enteroscopy was pursued, which revealed a bleeding jejunal mass. Staging with computed tomography (CT) of the abdomen/pelvis was negative for metastasis. Oncology recommended only surgical intervention with no chemotherapy. The patient successfully underwent a 16 cm laparoscopic small bowel resection and was discharged. On pathology, the resection localized a 2 × 1.5 × 0.2 cm tumor in the submucosa invading into the muscularis propria. The highly vascular tumor consisted of plump, polygonal tumor cells with pleomorphic nuclei. Giant cells were occasionally seen. Extravasated erythrocytes and irregular vascular channels were intermixed with the tumor cells. Endothelial marker, E26 transformation-specific related gene (ERG), showed diffuse positivity with high Ki-67 proliferative index (∼40%). Cytokeratin anion exchanger 1/anion exchanger 3 (AE1/AE3) revealed weak and focal positivity. The tumor cells were negative for smooth muscle activity, Melan-A, and S-100. The tumor's histomorphology and immunophenotype are consistent with angiosarcoma (Figure 3).

**Figure 4.**
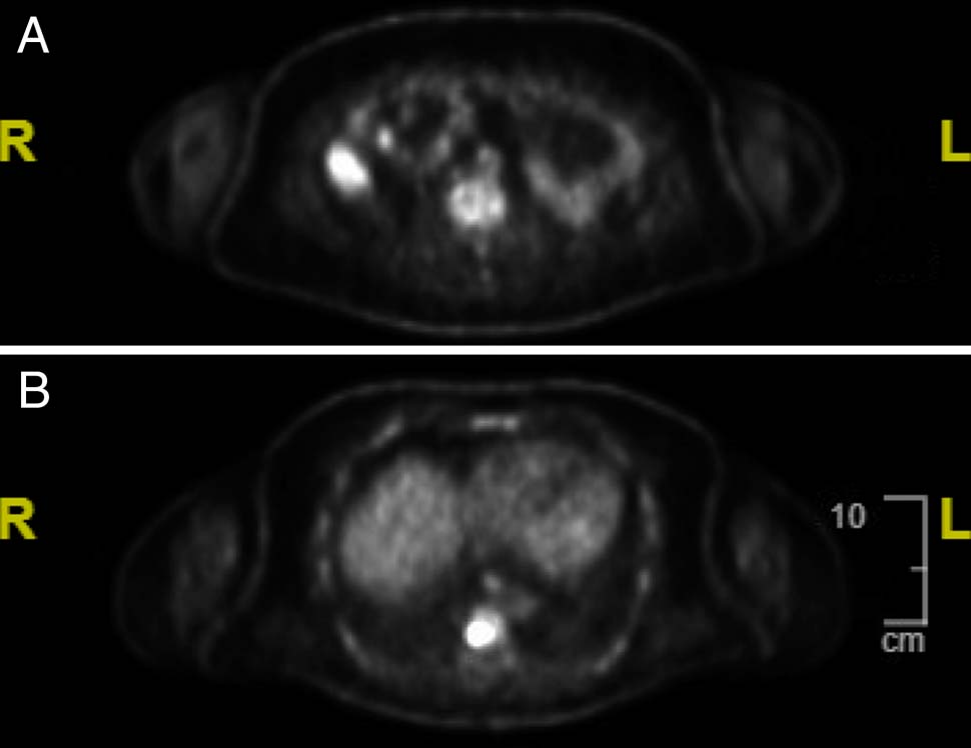
(A and B) Positron electron tomography imaging showing uptake in the vertebrae and small bowel, indicating metastasis post-small bowel resection.

Postsurgical follow-up 6 months later was significant for symptoms of anemia, dyspnea on exertion, and weakness all concerning for recurrence of his malignancy. A positron emission tomography (PET) scan was pursued which demonstrated multiple lesions in the small bowel and bone (Figure 4). The patient underwent 3.5 cm small bowel resection and began chemotherapy. Given his cardiac history, the decision was made to use paclitaxel instead of doxorubicin.

## DISCUSSION

Diagnosis of primary bowel angiosarcoma is rare and challenging. Cases of primary bowel angiosarcoma often present with nonspecific early gastrointestinal symptoms such as abdominal pain, anemia, gastrointestinal (GI) bleeding, fatigue/weakness, weight loss, dyspnea, dyspepsia, and nausea/vomiting.^2^ Diagnostic evaluation through esophagogastroduodenoscopy, capsule endoscopy, and colonoscopy is necessary to identify lesion locations and obtain tissue to confirm the diagnosis.^4–6^ However, involving immunohistochemistry markers may be necessary to confirm diagnosis and guide treatment plans.^7^ In previous studies, 23 of 62 small bowel angiosarcoma were detected using endoscopy alone.^13–15^ Alternative methods like CT and capsule endoscopy accounted for a smaller proportion of lesion detection. In our case, the tumor was localized by capsule endoscopy and the CT was unrevealing. Other imaging techniques such as CT angiography, magnetic resonance imaging, or PET scans could be considered. Once a mass is localized, rapid histopathological identification of angiosarcoma biomarkers such as CD31, CD34, factor VIII-related antigen, E26 transformation specific (ETS)-related gene, friend leukemia integration 1, and von Willebrand factor are necessary.^3^ Other antigens present in this particular case included cytokeratin, which has been controversial in its diagnostic relevance. Some studies found that exhibiting any cytokeratin immunoreactivity in angiosarcomas was rare, with only 3% of biopsies yielding cytokeratin immunoreactivity.^8^ Other gastrointestinal-specific angiosarcomas of the small bowel and colon/rectum were noted to be positive for cytokeratin AE1/AE3 (7/8), cytokeratin 7 (2/8), Cam5.2/cytokeratin 8 (5/8), and cytokeratin 19 (5/8).^9^ While cytokeratin studies can be helpful to confirm diagnosis, this result can lead to diagnostic confusion with carcinomas, especially when the angiosarcoma displays epithelioid morphology.^9–12^

In our case, no metastatic disease was identified at the time of surgical resection. However, within 6 months of surgery, PET scan revealed numerous lesions of metastatic angiosarcoma in the small bowel and bone. In a literature review of 62 primary small bowel angiosarcomas, 56.5% of patients had distant metastatic disease. One-year survival among these cases was 20.8%.^3^ Other sarcoma types and metastatic small bowel cancers report higher survival rates, despite a similarly aggressive disease courses. A study of 161 angiosarcoma cases by the French Sarcoma Group found a median survival of 3.4 years and a 5-year overall survival rate of 43%.^16^ Comparatively, metastatic small bowel adenocarcinomas have a poor prognosis with a median overall survival of approximately 12–20 months.^17^

Systemic therapy with paclitaxel was used as per the guidelines by the National Comprehensive Cancer Network written in 2024 for angiosarcoma. Other therapies such as bevacizumab and pazopanib are not classically used as first-line treatment. Studies on systemic therapy for metastatic or aggressive soft tissue sarcoma have reported first-line paclitaxel regimens to have a median overall survival of 10.2 months compared with 5.4 months with pazopanib.^18,19^ However, more recent discussion on combination chemotherapy in late-stage soft tissue sarcoma such as paclitaxel and gemcitabine or paclitaxel and pazopanib have promising results with increased progression-free survival (PFS).^18,20,23^ While retrospective analyses and phase II trials have demonstrated that weekly paclitaxel offers modest PFS benefits in patients with angiosarcoma—with median PFS ranging from 4 to 7.6 months—these studies often encompass heterogeneous patient populations and tumor sites and lack standardized post-treatment surveillance protocols. This gap highlights the necessity for prospective studies focusing on specific angiosarcoma subtypes, such as primary bowel angiosarcoma, to better understand treatment outcomes and inform clinical decision-making. Limited studies have been conducted on the benefit of neoadjuvant/adjuvant chemotherapy for primary small bowel angiosarcoma, but considerations into more aggressive therapy despite early primary angiosarcoma detection may be necessary.^21,22^ Future studies determining the efficacy of new drug interventions like monoclonal antibodies (bevacizumab) alone and/or in combination with standard regimens for soft tissue sarcoma chemotherapy (gemcitabine and docetaxel) may be a promising next step.

## DISCLOSURES

Author contributions: M. Min, S. Gupta, and K. Farrakhan were responsible for conception and design of the work. M. Mai and S. Gupta designed, drafted and edited the manuscript. S. Lu edited and provided pathology information. P. Akerman was the primary operator and assisted with case report conception and editing. M. Min is the article guarantor.

Financial disclosure: None to report.

Previous presentation: ACG Annual Scientific Meeting; October 24–29, 2025; Philadelphia, Pennsylvania.

Informed consent was obtained for this case report.

## ABBREVIATIONS:

AE1/AE3: anion exchanger 1/anion exchanger 3
CT: computed tomography
ERG: E26 transformation-specific related gene
ETS: E26 transformation-specific
GI: gastrointestinal
PET: positron emission tomography
PFS: progression free survival
